# Antiarthritic and Anti-Inflammatory Properties of *Cannabis sativa* Essential Oil in an Animal Model

**DOI:** 10.3390/ph17010020

**Published:** 2023-12-22

**Authors:** Hamid Kabdy, Hajar Azraida, Fatimzahra Agouram, Sara Oufquir, Jawad Laadraoui, Abdelmounaim Baslam, Abdelfatah Aitbaba, Meryem El Ouazzani, Loubna Elyazouli, Rachida Aboufatima, Stefania Garzoli, Abderrahman Chait

**Affiliations:** 1 Laboratory of Pharmacology, Neurobiology, Anthropology and Environment, Department of Biology, Faculty of Sciences Semlalia, University Cadi Ayyad, Marrakech 40000, Morocco; h.kabdy.ced@uca.ac.ma (H.K.); hazarazraida@gmail.com (H.A.); agouram.f2@gmail.com (F.A.); sara.oufquir@edu.uca.ac.ma (S.O.); baslamounaim@gmail.com (A.B.); a.aitbaba@gmail.com (A.A.); loubna.elyazouli@uca.ac.ma (L.E.); chait@gmail.com (A.C.); 2 Health and Environment Laboratory, Biochemistry, Biotechnology and Immunophysiopathology Research Team, Aïn Chock Faculty of Sciences, Hassan II University of Casablanca, Casablanca 20470, Morocco; jawad.laadraoui@edu.uca.ac.ma; 3 Anatomic Pathology Laboratory, FMPM-UCA-CHU Mohamed VI, Marrakech 40000, Morocco; meryem.el.ouazzani.88@gmail.com; 4 Laboratory of Genie Biologic, Faculty of Sciences and Technics, Sultan Moulay Slimane University, Beni Mellal 23040, Morocco; rachaboufatima@gmail.com; 5 Department of Chemistry and Technologies of Drug, Sapienza University, P. le Aldo Moro, 5, 00185 Rome, Italy

**Keywords:** chemical composition, inflammation, arthritis, in vivo experimentation

## Abstract

Arthritis and inflammatory conditions require effective therapies, but conventional drugs have side effects. This study explored *Cannabis sativa* L. essential oil (CSEO) as a safer alternative. A chemical characterization of EO conducted via GC/MS showed the presence of sesquiterpene hydrocarbons (67.63%), oxygenated sesquiterpenes (25.91%), and oxygenated monoterpenes (0.99%). The study used three established inflammation induction tests: xylene-induced ear swelling, carrageenan-induced paw inflammation, and inflammation in the paw induced by Freund’s complete adjuvant (CFA). Xylene triggered acute inflammation in the ear, while carrageenan-induced acute inflammatory responses through edema and immune-cell recruitment in the paw. CFA-induced arthritis simulated chronic inflammatory conditions. The obtained results demonstrated that treatment with CSEO significantly reduced ear weight in the xylene-induced ear-swelling test, indicating potential inhibition of neutrophil accumulation. In the carrageenan-induced paw inflammation test, CSEO reduced paw volume, suggesting interference with edema formation and leukocyte migration. In the CFA-induced paw inflammation test, CSEO decreased contralateral paw volume, restored body weight, and reduced C-reactive protein levels. Conclusion: this study provides compelling evidence supporting the antiarthritic and anti-inflammatory effects of CSEO. The findings indicate the therapeutic value of EO in the management of arthritis and inflammatory diseases while highlighting the need for further in-depth research to study the molecular mechanisms and validate their safety and efficacy for clinical applications. Preliminary data from this study suggests encouraging prospects for advancing the treatment and prevention of inflammation.

## 1. Introduction

Inflammation is the body’s natural and protective response to various stimuli, such as pathogens, injuries, or irritants, it is an innate and complex biological process characterized by several molecular and cellular events that work together to eliminate the cause of cell injury, clear out damaged cells and tissues, and initiate tissue repair [1]. This response serves as the body’s defense mechanism against tissue damage or the intrusion of external agents like toxins and pathogens [2]. The outcome and repercussions of the inflammatory response hinge significantly on the duration and extent of the reaction. The acute inflammatory process involves actions such as phagocytosis, apoptosis, or the activation of proinflammatory mediators, all aimed at eliminating harmful stimuli and restoring normal physiological conditions [3]. Nevertheless, persistent inflammation is counterproductive, giving rise to numerous pathological conditions such as rheumatoid arthritis [4].

Rheumatoid arthritis (RA) is a chronic, inflammatory autoimmune syndrome that damages joint cartilage and causes joint stiffness and immobility by triggering excessive, pus-free proliferation of the synovial membrane along with inflammation [5]. This condition affects approximately 0.3–1% of the world’s population and women are more prone to developing the disease than men [6]. RA predominantly affects older individuals and is linked to systemic complications, reduced quality of life, increased risk of premature death, and significant economic burdens [7].

It is characterized by inflammation of the synovial membrane, leading to swelling, the production of autoantibodies, and the deterioration of cartilage and bone. It is widely recognized that proinflammatory cytokines such as interleukins 1 (IL-1), interleukin 6 (IL-6), and tumor necrosis factor alpha (TNF-α) play a crucial role in promoting autoimmunity, persistent inflammation, and joint damage [8]. These cytokines are produced by macrophages and CD4+ T lymphocytes in the synovium. The increase in lining-layer cells of macrophage origin is prominent early in the disease process [9]. Numerous preclinical and clinical investigations have demonstrated increased levels of these cytokines in both blood serum and synovial fluid of individuals diagnosed with (RA) [10].

The primary approach to treating inflammation and pain involves the use of anti-inflammatory medications, such as nonsteroidal anti-inflammatory drugs (NSAIDs) and corticosteroids. However, NSAIDs can cause gastrointestinal issues, kidney problems, cardiovascular complications, skin reactions, and liver problems [11].

Consequently, it is necessary to explore the development of new drugs for the treatment of inflammation and pain [9,10]. Medicinal plants play a significant role in the prevention and treatment of human diseases. For thousands of years, people have used plants as traditional medicine [12]. The main plants with anti-inflammatory properties, as listed in the Pharmacopoeia and used for oral administration, include willow (*Salix purpurea*, *S. daphnoides*, *S. fragilis*, and *S. alba*), meadowsweet (*Filipendula ulmaria*), blackcurrant (*Ribes nigrum*), nettle (*Urtica urens*, *U. dioica*), ash (*Fraxinus excelsior*), harpagophyton (*Harpagophytum procumbens*, *H. zeyheri*), licorice (*Glycyrrhiza glabra*), and turmeric (*Curcuma longa*) [13]

*Cannabis sativa* is renowned for its recreational and therapeutic properties [14]. These plants contain a resin rich in terpenes, which they store in abundant glandular trichomes on the female flowers. This resin is composed of various groups of monoterpenes and sesquiterpenes, which play a fundamental role in defining the distinctive sensory characteristics of cannabis [14]. Furthermore, these compounds can influence the medicinal properties of different cannabis varieties [15]. The complexity of the chemical composition of cannabis has led to its traditional use in various medical and ethnobotanical applications [14]. Historically, *Cannabis sativa* has been used to relieve pain, treat rheumatic conditions, and soothe skin burns [16]. Additionally, recent research has demonstrated the effectiveness of cannabis extracts in reducing inflammation in skin fibroblasts [17], lung fibroblasts [16], and human tissues [18]. It is important to note that these terpenes possess well-established anti-inflammatory properties [18,19].

Additionally, studies have demonstrated that cannabidiol (CBD) contributes to alleviating intestinal inflammation and reducing thermal pain in animals, resulting in a decrease in the expression of inflammatory markers and inhibiting the production of IL-8 (interleukin-8, a proinflammatory cytokine) [20]. Other studies have shown that CBD reduces the circulating number of neutrophils and inhibits the release of inflammatory cytokines in mice treated with lipopolysaccharide, highlighting its anti-inflammatory role [21]. Beyond phytocannabinoids, over 150 terpenes have been identified in different cannabis cultivars. These compounds can contribute to various pharmacological properties of cannabis, including anti-inflammatory and antinociceptive effects [19]. Several studies have evaluated these effects in inflammation management, highlighting monoterpenes such as β-myrcene, α-pinene (inhibition of IL-6, TNF-α, and nitric oxide production and the suppression of MAPK and NF-κB activities in peritoneal macrophages) [20], limonene (inhibition of cytokines TNF-α, IL-1β, and IL-10 production), and linalool (regulation of K1, voltage-gated Na1, and Ca21 channels). Moreover, sesquiterpenes such as β-caryophyllene (reduction of the expression and production of proinflammatory cytokines, such as IL-1β, IL-6, IL-8, and TNF-α) [21] and α-humulene (downregulation of iNOS and COX-2 gene expression through the inhibition of NF-κB and AP-1 (ERK and p38) pathways) [19] have also demonstrated anti-inflammatory properties. These findings underscore the diversity of cannabis compounds and their potential in inflammation management. In light of the latter, we decided to evaluate the potential therapeutic properties of *Cannabis sativa* essential oil in the fight against acute and chronic inflammation. The novelty of our study lies in the unique nature of the extract; as of this date, no other study has demonstrated the anti-inflammatory effects of the essential oil of Cannabis sativa on various types of inflammation. To do this, we used well-established acute and chronic assays to induce inflammation, including xylene-induced ear swelling, carrageenan-induced paw inflammation, and complete paw inflammation triggered by Freund’s adjuvant (CFA).

## 2. Results

### 2.1. Chemical Composition of CSEO

A GC-MS analysis of CSEO allowed for the identification of 32 components, corresponding to 96.94% of its total chemical composition (Table 1). The detected chemical classes were sesquiterpene hydrocarbons (67.63%), oxygenated sesquiterpenes (25.91%), and oxygenated monoterpenes (0.99%). €-Caryophyllene (41.59%), α-humulene (14.93%), and caryophyllene oxide (11.4%) were the main constituents of CSEO.

### 2.2. Anti-Inflammatory Activity

#### 2.2.1. Xylene-Induced Ear Oedema

**Figure 1:**
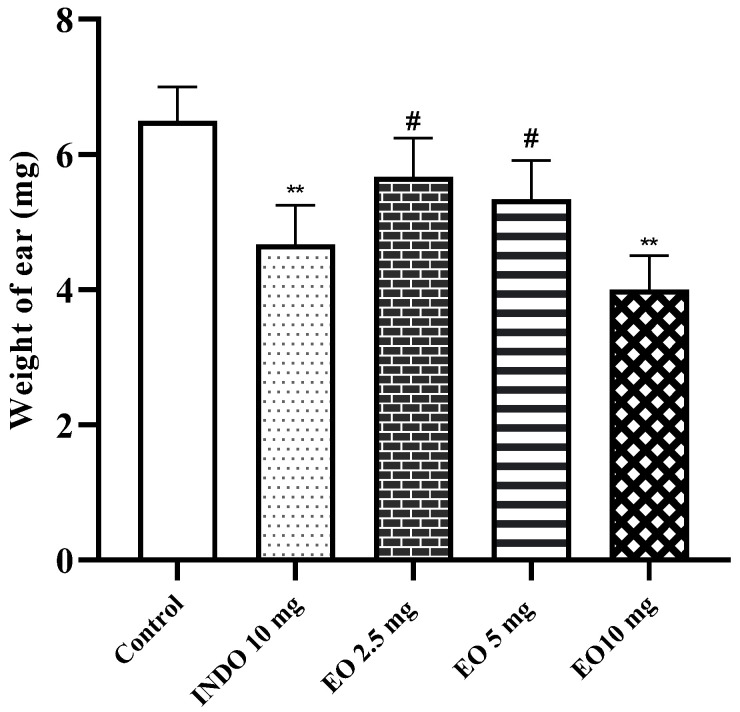
Effect of CSEO on xylene-application-induced ear edema in mice. The graph shows the difference between the left and right ear. INDO: Indomethacin, EO: Essential oil. Results are presented as mean ± SEM (n = 6). ‘**’ vs. Control, ‘#’ vs. EO 10 mg ‘#’: *p* < 0.05; ‘**’ *p* < 0.01.

Application of xylene to the right ears of mice resulted in a visible increase in edema compared to the left ears. The weight of the right ear was compared to that of the left ear of the same animals. Treatment with CSEO (2.5, 5, and 10 mg/kg) resulted in a significant reduction (*p* < 0.01) in-ear edema (Figure 1), with a significant dose-dependent effect with 10 mg (*p* < 0.05). Similarly, indomethacin also significantly reduced ear edema.

#### 2.2.2. Histological Examination

**Figure 2:**
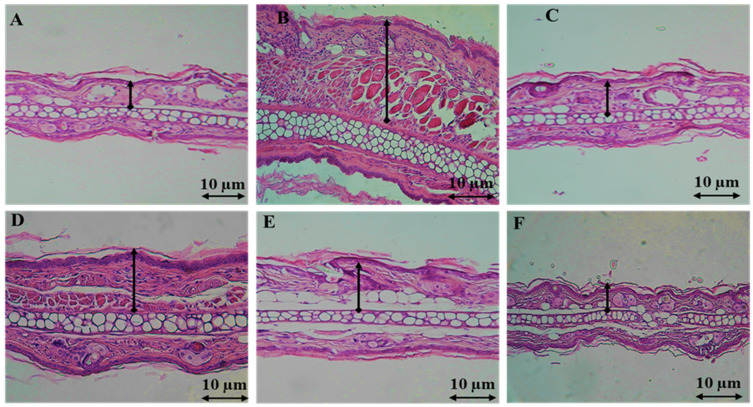
Tissue sections indicating the swelling of the ear caused by xylene treatment. (**A**) The normal ear; (**B**) model control group induced by xylene; (**C**) the treatment with indomethacin (10 mg/kg); (**D**) 2.5 mg/kg CSEO, (**E**) 5 mg/kg CSEO, (**F**) 10 mg/kg CSEO. Sections were stained with H&E (×100). Scale bar = 10 µm.

Ear sections from mice with xylene-induced ear edema showed the presence of various acute inflammatory cells, such as neutrophils, plasma cells, and lymphocytes. The connective tissue stroma was filled with edema-forming fluid, while the dermal lining appeared normal. However, treatment with CSEO containing essential oil of Cannabis sativa at a dose of 10 mg/kg showed a significant anti-inflammatory effect. This effect was observed at the cellular level, with a decrease in the density of fibrous connective tissue and collagen. The cartilage tissue appeared normal. In comparison, indomethacin, the reference drug, showed a decrease in acute inflammatory cellular infiltrates in the sparse stromal region. The nuclei of the epithelial lining of the dermis appeared normal (Figure 2).

#### 2.2.3. Carrageenan-Induced Rat-Paw Edema

The anti-inflammatory effect of CSEO was evaluated using the paw edema model. Treatment with CSEO at doses of 2.5, 5, and 10 mg/kg significantly reduced (*p* < 0.05) carrageenan-induced edema formation, with this effect being observed at 2, 3, and 4 h after administration of the phlogistic suspension. Furthermore, the 10 mg/kg dose showed a significant inhibition percentage. Similarly, IND at a dose of 10 mg/kg also significantly inhibited (*p* < 0.001) the edematogenic response evoked by carrageenan in rats at 1, 2, 3, and 4 h. These findings are summarized in Table 2.

### 2.3. Adjuvant-Induced Chronic Arthritis

#### 2.3.1. Body-Weight Changes in CFA-Induced Arthritic Rats

During the initial evaluation (day 0 before immunization), the body weight was almost equivalent in all groups of animals. However, throughout the subsequent period, the body weights of immunized rats remained consistently lower than those of the control rats. Towards the end of the last week, a trend was observed among immunized rats to regain a weight similar to that of the control rats.

#### 2.3.2. Anti-Inflammatory Activity of EOCS in CFA-Induced Arthritic Rats

**Figure 3:**
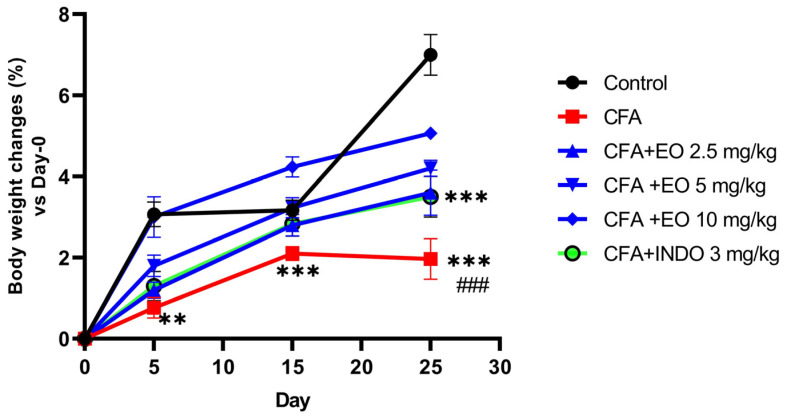
The effect of CSEO on body-weight changes within an arthritis-induced model. Results are presented as mean ± SEM (n = 6). CFA: Freund’s complete adjuvant, INDO: indomethacin, EO: essential oil. Statistical analyses were done according to two-way ANOVA, followed by Tukey’s post hoc test, # vs. CFA. ‘**’ *p* < 0.01; ‘***’ or ‘###’ *p* < 0.001.

**Figure 4:**
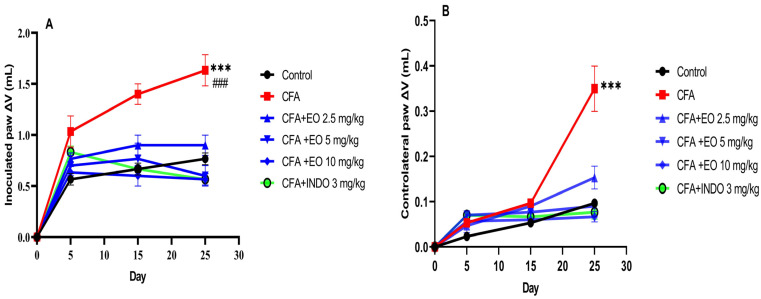
Anti-inflammatory effect of CSEO on arthritis-induced model. (**A**) Inoculated paw volume changes. (**B**) Contralateral paw volume changes. CFA: Freund’s complete adjuvant, INDO: indomethacin, EO: essential oil. Results are presented as mean ± SEM (n = 6). Statistical analyses were done according to two-way ANOVA, followed by Tukey’s post hoc test, ‘***’ vs. vehicle; ### vs. CFA. ‘***’ or ‘###’ *p* < 0.001.

By subjecting the changes in paw volume of the injected paw (right) to repeated measures ANOVA, the results revealed significant effects attributed to the treatment groups (*p* < 0.001), time (*p* < 0.001), and a significant interaction between treatment and time (*p* < 0.001), as shown in Figure 3. A post hoc analysis using the Newman–Keuls test indicated a notable increase in paw volume in all groups that received the CFA injection compared to the control group (*p* < 0.05). Both the groups treated with Cannabis sativa essential oil and the CFA + indomethacin group demonstrated a significant reduction in paw volume (*p* < 0.001) when compared to the CFA group (Figure 4).

#### 2.3.3. Effect of EOCS on % Inhibition of Arthritis in CFA-Induced Arthritic Rats

**Figure 5:**
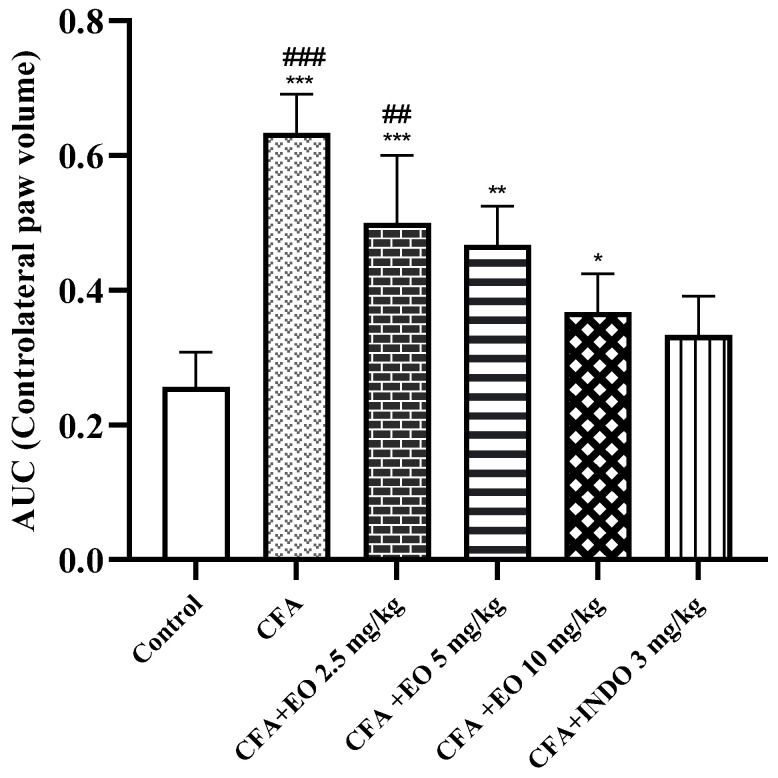
Effect of CSEO on inhibition of arthritis development in a model. Results are presented as mean ± SEM (n = 6). CFA: Freund’s complete adjuvant, INDO: indomethacin, EO: essential oil. Statistical analyses were done according to two-way ANOVA, followed by Tukey’s post hoc test, ‘*’ vs. vehicle, *p* < 0.05; ‘**’ or ‘##’ *p* < 0.01; ‘***’ or ‘###’ *p* < 0.001.

Indomethacin administration demonstrated significant efficacy in reducing paw volume (*p* < 0.001) compared to the CFA group, while a notable trend towards anti-inflammatory effects was observed at the highest dose of CSEO (10 mg/kg) (*p* > 0.001). Importantly, no substantial anti-inflammatory activity was observed in CFA-induced rats treated with the lower dose of CSEO (2.5 mg/kg). When the data, represented as area under the curve (AUC) for the volume of the contralateral paw over time, was analyzed, and the percentage of inhibition was calculated relative to the arthritic control rats (CFA group), it was evident that the indomethacin consistently reduced paw swelling throughout the study (*p* < 0.001 vs. CFA group). In contrast, CSEO at the highest dose (10 mg/kg) exhibited a trend towards efficacy (*p* > 0.001 vs. CFA group), as illustrated in Figure 5.

#### 2.3.4. Effect of EOCS on the Arthritis Score in CFA-Induced Arthritic Rats

**Figure 6:**
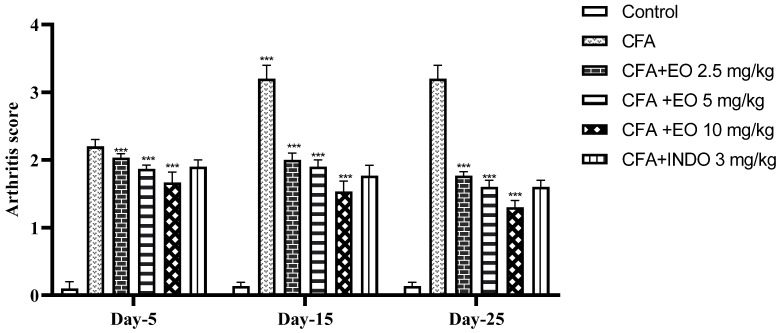
Effect of CSEO on arthritis score in arthritis-induced model CFA. Results are presented as mean ± SEM (n = 6). CFA: Freund’s complete adjuvant, INDO: indomethacin, EO: essential oil. Statistical analyses were done according to two-way ANOVA, followed by Tukey’s post hoc test, ‘***’ vs. vehicle; ‘***’ *p* < 0.001.

The mobility assessment was subjected to a two-way repeated measures ANOVA, revealing significant effects associated with treatment (*p* < 0.001), time (*p* < 0.001), and the interaction between treatment and time (*p* < 0.001), as illustrated in Figure 6. A Newman–Keuls post hoc analysis distinctly demonstrated a notable increase in arthritis scores in all CFA-inoculated groups compared to the control group (*p* < 0.05). Although no significant distinctions were noted between the CFA-inoculated groups (*p* > 0.05), changes became evident starting from on day 15, with an increase in arthritis scores in the CFA group and a decrease in the treated groups, depending on the administered dose.

The administration of indomethacin significantly reduced arthritis scores (*p* < 0.001), demonstrating a decrease compared to the CFA group.

#### 2.3.5. Effect of EOCS on Plasma CRP Levels in CFA-Induced Arthritic Rats

For plasma CRP levels, a one-way ANOVA revealed significant treatment effects on day 15 (*p* < 0.001), as shown in Table 3. CRP levels in the CFA + EOCS groups were significantly higher than those measured in all groups on day 15, while they returned to normal values (such as in healthy control rats) on day 25 (*p* > 0.001).

## 3. Discussion

Arthritis and inflammatory conditions pose significant health challenges, requiring effective therapeutic interventions. Current clinical management involves nonsteroidal and steroidal anti-inflammatory drugs. However, prolonged use of these drugs often leads to serious side effects, in particular renal problems, gastrointestinal irritations, and ulcerations [22]. Hence, the imperative to discover potent anti-inflammatory molecules without these limitations remains unmet, prompting research into medicinal plants used in complementary and traditional medicine to relieve pain, fever, and rheumatic disorders [23].

In this investigation, our specific goals centered on evaluating the potential antiarthritic and anti-inflammatory properties inherent in *Cannabis sativa* essential oil. To achieve this, we employed established models for inducing inflammation, including xylene-triggered ear swelling, carrageenan-induced paw inflammation, and complete Freund’s adjuvant (CFA)-induced paw inflammation. These chosen assays were designed to scrutinize the capacity of Cannabis sativa essential oil to alleviate inflammatory responses, with a particular emphasis on its potential effectiveness against arthritis and inflammation. The selection of Cannabis sativa essential oil was based on its documented bioactive compounds, notably cannabinoids, which have exhibited promise in modulating inflammatory pathways. Furthermore, the historical utilization of Cannabis sativa for its anti-inflammatory properties in traditional medicine provided a rationale for exploring its therapeutic potential in the context of arthritis and inflammation.

The xylene-induced ear inflammation test serves as a validated model for assessing acute inflammation triggered by xylene, which induces various inflammatory mediators [24]. Xylene-induced edema is, in part, associated with substance P release. Sensory neurons, primarily responsible for substance P release, enhance vessel permeability, leading to plasma extravasation and neurogenic inflammation. This process also involves the release of inflammatory mediators such as histamine, fibrinolysins, and kinins [25].

Our findings indicate that the administration of *Cannabis sativa* essential oil (CSEO) significantly decreased ear weight, particularly at the 10 mg/kg dose, in response to the initially increased ear edema induced by xylene. Indomethacin, a reference drug, similarly demonstrated a significant reduction in ear edema at 10 mg/kg. The observed weight gain and inflammation in the ear can be attributed to neutrophil accumulation, a key factor in inflammatory skin conditions like dermatitis and is a critical aspect of the disease’s pathological mechanism [26]. Histological analysis further affirmed the oil’s inhibitory effect on inflammation, potentially attributed to its modulation of proinflammatory mediators, such as cytokines and prostaglandins.

The anti-inflammatory effect of CSEO at a 10 mg/kg dose, was significant, as evidenced at the cellular level by a reduction in fibrous connective tissue and collagen density, while cartilage tissue appeared normal. In comparison, indomethacin exhibited a decrease in acute inflammatory cellular infiltrates in the sparse stromal region, with the nuclei of the epithelial lining of the dermis appearing normal.

The carrageenan-induced paw inflammation test, widely acknowledged for assessing the efficacy of anti-inflammatory agents, elicits acute inflammatory responses characterized by edema and the recruitment of immune cells at the injection site [27]. Carrageenan-induced edema is a two-phase process widely utilized to assess the anti-inflammatory efficacy of novel agents. This edema formation, triggered by carrageenan, engages multiple mediators such as prostaglandins, vasoactive amines, bradykinin, and leukotrienes. Inhibiting the synthesis and release of these mediators effectively diminishes pain and inflammation. The initial phase, lasting 1–2 h, involves the release of histamine and serotonin, while the subsequent phase is linked to the liberation of leukotrienes and prostaglandins [28].

Our results indicate a notable reduction in paw volume following the administration of Cannabis sativa essential oil (CSEO) at doses of 2.5, 5, and 10 mg/kg, particularly evident four hours after carrageenan injection. The observed anti-inflammatory effects likely stem from the interference with the signaling pathways responsible for edema formation and the migration of leukocytes. Furthermore, the essential oil may exert its inhibitory effects on enzymes, such as cyclooxygenases and lipoxygenases, thus contributing to the attenuation of inflammation in this model. This dual-action mechanism, involving modulation of signaling pathways and enzyme inhibition, underscores the comprehensive nature of CSEO’s anti-inflammatory potential in the context of carrageenan-induced paw inflammation.

The CFA-induced paw inflammation assay serves as a model to replicate chronic inflammatory conditions. CFA-induced arthritis develops in two phases: an initial acute phase, starting from day 0, characterized by local inflammatory reactions leading to primary arthritic manifestations such as paw edema, followed by a subsequent chronic inflammatory phase that emerges around day 15. This chronic phase is distinguished by inflammatory edema in the contralateral paw [29]. The initial acute phase is triggered by various mediators, including cytokines such as IL-1β and TNF-α, prostaglandins, histamine, kinins, and serotonin released by leukocytes migrating to the affected area, resulting in edema [30]. The chronic phase involves cell-mediated autoimmunity, with the cytokine IL-6 playing a crucial role in the processes that lead to the degeneration of cartilage and bone. The nociceptive responses induced by CFA are probably linked to its ability to interrupt cellular signaling pathways, particularly those involving PKC, NO, Ca^2+^, and K^+^ [31].

The administration of CSEO resulted in a significant reduction in contralateral paw volume at all doses (2.5, 5, and 10 mg/g), similar to indomethacin, which exhibited a similar effect at 3 mg/kg and led to the restoration of body weight in mice. The body weights of mice treated with doses of 2.5, 5, and 10 mg/kg remained consistently lower than that of the control group. On the 15th day, a trend was observed among treated mice to regain a weight similar to that of the control mice. Regarding arthritis scores, changes became evident from the 15th day, with an increase in arthritis scores in the CFA group up to 3.4 and a decrease in the treated groups, especially the group treated at 10 mg/kg. Administration of indomethacin significantly reduced arthritis scores, demonstrating a decrease compared to the CFA group. Thanks to treatment with CSEO at all doses (2.5, 5, and 10 mg/kg), plasma CRP levels returned to normal by day 25, implying potential anti-inflammatory and immune-modulatory properties [32]. The observed connection between the extent of joint inflammation and weight loss within the CFA group highlights the potential of the EO to alleviate chronic inflammation [33]. Furthermore, the reduction in C-reactive protein (CRP) levels provides additional evidence supporting the anti-inflammatory potential of the EO, considering the well-established role of CRP as an inflammation biomarker [34].

The effect exerted by CSEO can be justified through various mechanisms; for instance, during exposure to inflammatory agents, free radicals may also be generated [35]. By acting as an antioxidant [36], the essential oil could neutralize these free radicals, thereby mitigating inflammation and the associated cellular damage [37]. Alternatively, the essential oil may possess the ability to modulate the activation and accumulation of immune cells, such as neutrophils, influencing the nature and intensity of the observed inflammation [38]. It may also reduce vascular permeability [39], inhibit gene expression by altering the production of proteins involved in the inflammatory response [40], or modulate receptors by interacting with them [41]. The compounds in the essential oil could thereby modulate the cellular response to inflammation induced by xylene, carrageenan, or CFA.

Previous research consistently affirms the anti-inflammatory potential of CSEO. This was exemplified by Rossi and colleagues [33], who specifically demonstrated the reduction in cytokine release induced by etoposide on cutaneous cell lines through hemp EO [33]. Moreover, specific constituents of hemp EO, such as (E)-caryophyllene and α-humulene, have been identified as contributors to in vivo anti-inflammatory effects, effectively decreasing the release of IL-1β and TNF-α [34,35].

In relation to this, another study highlighted the different anti-inflammatory and antinociceptive effects of the EOs rich in terpenoids from cannabis chemotypes. This research highlighted that, independent of TNFα, these EOs exhibit significant anti-inflammatory properties. Moreover, the study underlined that purified CBD is more effective than EOs, providing sustained immunosuppression for chronic inflammation, while terpenoids offer transient immunosuppression, which is potentially useful for alleviating acute inflammation [30]. Similarly, another study showed significant inhibition of the inflammatory response in chronic and acute rat inflammation models through the use of EO extract from Lebanese *Cannabis sativa* L. ssp. indica (Lam.) at doses of 25 and 50 mg/kg [31].

Moving to a related research path, the acyclic diterpene phytol from hemp-seed EO was found to prevent macrophage activation and modify macrophage plasticity in human tissue under inflammatory conditions [36]. Moreover, β-caryophyllene, a major constituent of *Cannabis sativa*, presented anti-inflammatory effects through oral administration. This component significantly reduced spinal neuroinflammation in animal models [37] and improved colitis via the activation of CB2 receptors [38]. Additionally, *Cannabis* extracts with high levels of delta-9-tetrahydrocannabinol have been found to effectively reduce inflammation by reducing cytokine expression [17].

Further strengthening these findings, several studies using highly CBD-enriched Cannabis sativa extracts demonstrated a substantial inhibition of inflammation. These studies recorded inhibitions of 43% and 64% at 25 mg and 50 mg/kg body weight, respectively, in a mouse-paw edema model induced by zymosan after 24 h [30]. Moreover, a petroleum ether extract from *Cannabis sativa* seeds induced a reduction in edema size, with inhibitions of 57.22% and 92.32% at 6 and 24 h, respectively, in carrageenan-induced paw edema in rats [39].

The results of this collective research highlight the significant anti-inflammatory potential of *Cannabis sativa* and its various components, underscoring their potential therapeutic applications in managing inflammatory conditions. This explains the need to explore alternative therapies, such as *Cannabis sativa*, due to their potential effectiveness, reduction of side effects, holistic approach, personalized treatments, exploration of new therapeutic targets, cultural acceptability, and symptom palliation.

However, clinical studies are crucial to establishing their efficacy and safety, taking into account individual variability in treatment response, including genetic and environmental factors that could be examined. This individual variability may be one of the factors influencing the results of this study, as well as the limited number of animal samples per group, constituting a limiting factor. The duration of the arthritis study could also be a potential limitation, with 25 days possibly not covering all the relevant temporal aspects.

Our study provides compelling evidence of the antiarthritic and anti-inflammatory effects of CSEO. Through rigorous testing, we have demonstrated its ability to inhibit acute and chronic inflammation, emphasizing its potential therapeutic value in managing arthritis and inflammatory conditions. Further research is needed to validate the safety and efficacy of CSEO for potential clinical applications, including the testing of inhibition of inflammatory mediator release and measuring levels of inflammatory mediators, such as TNF-alpha, IL-1beta, and IL-6, in tissues or serum using immuno-enzymatic techniques (ELISA) [42,43]. Studying cytokines in synovial fluid is also necessary to obtain more specific information on local changes [9], as well as to evaluate joint function using functional tests to assess joint mobility in laboratory animals, such as the mechanical allodynia test or the knee joint test. Furthermore, additional research is needed to deepen our understanding of molecular mechanisms by exploring specific cellular signaling pathways associated with the anti-inflammatory properties of the essential oil.

## 4. Materials and Methods

### 4.1. Plant Material

*Cannabis sativa* EO from leaves collected in the Rif region of northern Morocco (34°54′57″ N, 4°34′07″ W) was obtained by a hydrodistillation process using a Clevenger apparatus. The aerial parts of *Cannabis sativa* were gathered in the Rif region located in northern Morocco (34°54′57″ N, 4°34′07″ W). Professor A. Ouhammou of the Laboratory of Environment and Ecology identified the plant material, which was subsequently deposited in the Herbarium of the Faculty of Sciences Semlalia, Cadi Ayyad University, Marrakech, Morocco (Voucher number: MARK-14366). *Cannabis sativa* leaves underwent a four-hour hydrodistillation process using a Clevenger apparatus, and this procedure was repeated several times to guarantee an ample essential oil yield. This method entails heating water in a flask and passing the resulting steam through the aromatic plant material, carrying the essential oils along. The steam is subsequently condensed in a cooler (freezer temperature), resulting in a blend of water and essential oil. The obtained essential oil is then stored in a light-protected environment at a temperature of 4 °C.

### 4.2. Gas Chromatography–Mass Spectrometry (GC/MS) Analysis

Gas chromatography–mass spectrometry analysis was performed by using a Thermo Fisher Scientific (Waltham, MA, USA) ISQ 1712507 1300 tracer equipped with a flame ionization detector and a single quadrupole mass spectrometer. One µL of the sample was injected into an Rtx-5 column (serial number: 952770). The column temperature was maintained at 35 °C for 5 min and then increased to 250 °C at a rate of 5 °C/min. The operative conditions of the mass spectrometer were as follows: carrier-gas flow rate of 1 mL He/min and ion source and detection temperatures of 200 °C and 250 °C, respectively. The electron impact mode was set at 70 Ev with a scan mass range of m/z 40–450. The identification of the components was carried out by determining their retention indices (RI) using a homologous series of n-alkanes (C_9_–C_24_) and comparing their mass spectra with reference libraries.

### 4.3. Animals

The experiments described in the passage were carried out on adult male Swiss albino mice and Sprague–Dawley rats. These animals were raised at the Cadi Ayyad University animal facility in Semlalia Marrakech, Morocco, and housed six to a cage with ad libitum access to food and water. The mice were maintained under controlled lighting conditions with a 12/12 h light–dark cycle and a temperature of 20 ± 2 °C. Throughout the experiments, which followed the ethical guidelines for experimental studies with conscious animals (86/609/EEC) and, strict adherence to the ARRIVE guidelines was maintained, ensuring comprehensive reporting standards.

### 4.4. Drugs and Treatments

A concentration of 10 mg/mL of Freund’s complete adjuvant (CFA) was obtained from Sigma Aldrich (St. Louis, MI, USA). Indomethacin (IND) was administered at a dose of 10 mg/kg. The negative control group was treated with the vehicle Tween 80 (10 mL/kg) +0.9%. Xylene and carrageenan were obtained from Sigma Aldrich. Doses (2.5, 5, and 10 mg/kg) of *Cannabis sativa* essential oil (EOCS) are administered intraperitoneally.

### 4.5. Xylene-Induced Ear Oedema

Xylene-induced ear edema was performed according to the protocol previously described by Akindele and Adeyemi [40]. Briefly, each group of mice received intraperitoneal injections of CSEO at doses of 2.5, 5, and 10 mg/kg, while the control group received distilled water. Indomethacin (10 mg/kg) was used as a positive control. The left ear was considered an intact negative control, while the inner and outer surfaces of the right ear were treated with 0.02 µL of xylene. One hour after the xylene application, animals (six per group) were sacrificed by cervical dislocation in accordance with ethical standards. The right and left ears were isolated, and 5 mm sections were taken and weighed. Ear edema was assessed by calculating the weight difference between the right and left ear sections from the same animal. Ear sections were fixed in 10% formalin and cut into 4 µm thick sections, which were stained with hematoxylin and eosin for analysis.

The percentage of edema inhibition was calculated according to the following relationship: inhibition (%) = difference in ear weight (control)/difference in ear weight (treat)/difference in ear weight (control) × 100%.

### 4.6. Carrageenan-Induced Rat-Paw Edema

Male Sprague–Dawley rats weighing 160–200 g were fasted for 18 h before the experiment. They were divided into groups and received CSEO at doses of 2.5, 5, and 10 mg/kg, or indomethacin at a dose of 10 mg/kg as a positive control. The paw thickness was measured using a caliper from the ventral to the dorsal surface of the paw, 1 h before injecting 0.1 mL of carrageenan (1% suspension in 0.9% saline) into the subplantar area. The paw thickness was then measured every hour for 4 h.

The degree of edema was calculated by comparing the increase in paw thickness after carrageenan injection with the paw thickness measured before injection for each rat. The results were expressed as the percentage of edema inhibition in the treated groups compared to the control group.

### 4.7. CFA-Induced Arthritis Model

Using hydrate chloride, the animals were anesthetized, and arthritis was triggered by a single intradermal injection of CFA (0.01 mL) into the posterior right metatarsal pad. The control group rats were anesthetized and received an injection of an equivalent volume of saline solution instead of CFA. They were subsequently treated intraperitoneally with saline solution once a day for 25 days. The CFA-treated rats were divided into four groups and underwent daily treatment for 25 days. The first group received the CFA + EOCS treatment at a dose of 2.5 mg/kg, the second group received the CFA + EOCS treatment at a dosage of 5 mg/kg, the third group received the CFA + EOCS treatment at a dose of 10 mg/kg, while the fourth group was administered indomethacin at a dose of 3 mg/kg as a reference drug. Indomethacin solution was prepared by dissolving the powder in a saline solution. All treatments were administered 1 h before arthritis induction on day 0 and 1 h before testing on subsequent days.

### 4.8. Body-Weight Assessment

The body weights of the rats were assessed on days 0, 5, 15, and 25, and the results were presented as a percentage of the change from the initial weight (day 0).

### 4.9. Hind-Paw Volume Assessment

The paw sizes for both the injected hind paw and the contralateral hind paw were measured. (measured up to the hairline) were assessed on days 0 (prior to CFA immunization), 5, 10, 15, 20, and 25 using a plethysmograph. The calculation of edema or increase in paw volume (DV) at a specific time point (t) was determined using the following formula: paw volume (t0)—paw volume (t), with paw volume (t0) established on day 0 of first immunization. Before volume measurements, the animals were anesthetized with hydrate chloride.

### 4.10. Percentage Inhibition of Paw Edema

An inflammatory response, characterized by initial arthritic symptoms, occurred rapidly in the injected hind paw (right) within 30 min of injection and persisted for approximately 20 to 25 days. At the same time, secondary arthritic symptoms typically developed in the noninjected (left) paw approximately 15 days after sensitization, often lasting for a similar duration of 25 days. To evaluate the potential of *Cannabis sativa* essential oil treatment to mitigate or decrease the onset of these secondary arthritic symptoms compared to arthritic rats in the CFA group, we examined contralateral paw-volume data by calculating the area under the curve (AUC) from day 0 to day 25. Means were determined for each group, and the percentage of inhibition in paw swelling relative to the CFA group was calculated using the formula % inhibition of paw swelling = (B − A)/A × 100, where A represents the mean of the healthy control group minus the average of the CFA group and B represents the mean of the treated CFA group minus the average of the CFA group. This approach takes into account for nonzero values in the healthy control group. A percentage of inhibition close to 100% indicates the effectiveness of the treatment in preventing or reducing the incidence of arthritis compared to the arthritic CFA group.

### 4.11. Arthritis Score

The assessment of arthritis severity involved visual observations of walking ability on days 5, 15, and 25. We used a modified version of a previously reported scale [28]. Each animal was scored on a scale ranging from 0 to 4, where: 0 = the rat exhibited normal walking and running; 1 = the rat showed difficulty while walking and running; 2 = the rat showed limping without retracting the hind paw; 3 = the rat exhibited lameness with hind paw retracted (hind paw not touching the ground); and 4 = the rat moved exclusively by crawling or lying down. The maximum possible score for each arthritic rat was 4.

### 4.12. CRP Analysis

On the 25th day of the experiment, blood samples were taken from the tail veins of previously anesthetized animals using hydrated chloride. The blood was then placed into tubes containing ethylenediaminetetraacetic acid (EDTA) to prevent clotting and immediately subjected to centrifugation at 2000× *g* for 5 min. The resulting plasma was carefully collected and preserved at −80 °C for the subsequent analysis of CRP (C-reactive protein) concentrations.

### 4.13. Statistical Analysis

The findings were presented as the mean ± standard error of the mean and assessed using GraphPad Prism 8 statistical software. Prior to analysis, the normality of the data distribution was assured through the Shapiro–Wilk test, confirming adherence to a normal distribution. Subsequently, data analysis involved conducting one-way analysis of variance (ANOVA). Statistical significance was determined when the *p*-value was below 0.05 (*p* < 0.05) for all evaluations. This comprehensive approach ensured the validity of the parametric analysis methods used in this study.

## 5. Conclusions

In summary, the study conducted on *Cannabis sativa* essential oil presents robust evidence supporting its potent antiarthritic and anti-inflammatory effects. The research employed established inflammation induction tests, including xylene-induced ear swelling, carrageenan-induced paw inflammation, and complete Freund’s adjuvant (CFA)-induced paw inflammation. The results consistently demonstrated a significant reduction in inflammation, reaffirming the therapeutic potential of CSEO. While the study provides compelling evidence, further research is needed to delve deeper into the precise molecular mechanisms underlying the observed effects. Furthermore, rigorous validation of the safety and efficacy of CSEO in clinical settings is essential before considering its widespread therapeutic applications. Nonetheless, the results of this study contribute significantly to the growing body of evidence supporting the potential of CSEO in the management of arthritis and inflammatory conditions. Additional studies will be necessary to focus on elucidating specific molecular pathways, optimizing dosage regimens, and conducting comprehensive safety assessments. This approach will pave the way for the eventual integration of Cannabis sativa essential oil into mainstream strategies for managing inflammatory diseases.

## Figures and Tables

**Table 1:** Chemical composition (peak-area percentage) of *Cannabis sativa* essential oil (CSEO).

N˚	Compounds ^a^	RI Exp ^b^	RI Lit ^c^	%
1	α-Pinene	926	926	0.99
2	Borneol	941	939	0.11
3	L-*α*-Terpineol	971	968	0.17
4	Cubebol	999	992	1.82
5	Isocaryophyllene	1020	1012	0.69
6	*α*-Longipinene	1031	1027	0.27
7	(*E*)-Caryophyllene	1040	1032	41.59
8	2-Norpinene	1061	1053	0.99
9	Guaia-1(10),11-diene	1074	1065	0.31
10	*α*-Humulene	1103	1094	14.93
11	Epi-*β*-Caryophyllene	1132	1123	2.25
12	*β*-Patchoulene	1157	1148	0.21
13	α-Guaiene	1161	1152	0.52
14	Selina-3,7(11)-diene	1330	1320	0.40
15	*β*-Selinene	1344	1334	1.71
16	*α*-Selinene	1391	1382	1.41
17	1H-Benzocycloheptene	1418	1408	0.19
18	Farnesyl bromide	1425	1415	0.07
19	Patchoulene	1439	1429	0.10
20	δ-Cadinene	1450	1439	0.46
21	Selina-3,7(11)-diene	1466	1456	2.83
22	Caryophylladienol II	1482	1470	3.87
23	Cholecalciferol	1490	1478	0.10
24	Isolongifolol	1500	1483	0.27
25	Caryophyllene oxide	1507	1495	11.40
26	Ledol	1513	1501	0.69
27	Humulene oxide II	1527	1514	3.78
28	Costol	1537	1524	0.08
29	*γ*-costol	1550	1536	0.13
30	Aristol-1(10)-en-9-ol	1558	1543	0.31
31	Germacra-4(15),5,10(14)-trien-1α-ol	1581	1581.89	3.65
32	*α*-Bisabolol	1597	1597.64	0.64
	Total			96.94
	Oil yeld (%, *w*/*w*)			1.20
	Hydrocarbons Monoterpene (H.M.)			0.99
	Oxygenated Monoterpenes (O.M.)			0.47
	Hydrocarbon Sesquiterpenes (H.S.)			67.63
	Oxygenated Sesquiterpenes (O.S.)			25.91

^a^ The components are reported according to their elution order on an apolar column; ^b^ Linear retention indices measured on an apolar column; ^c^ Linear retention index taken from Adams (2007) or NIST 08 (2008), FFNSC 2 (2012), and the literature (for compound 32).

**Table 2:** Effect of CSEO and indomethacin on carrageenan-induced dermal paw edema in rats. The table shows the paw volume tracking after carrageenan administration for 1 h, 2, 3, and 4 h.

		Mean Paw Volume ± SD (mL) (% Inhibition)				
Treatment		0 h	1 h	2 h	3 h	4 h
**Control**	Paw volume	1.64 ± 0.05	1.74 ± 0.04	2.16 ± 0.07	0.1 ± 0.04	3.62 ± 0.04
	Inhibition	-	-	-	-	-
**Indomethacin** **10 mg/kg bw**	Paw volume	1.56 ± 0.05	1.43 ± 0.03	1.62 ± 0.01	1.67 ± 0,02	1.84 ± 0.04
	Inhibition	4.87804878	17.81609195	25.00	46.12903226	49.1713 *
**EOCS** **2.5 mg/kg bw**	Paw volume	1.45 ± 0.10	1.92 ± 0.02	2.06 ± 0.03	2.93 ± 0.07	3.12 ± 0.07
	Inhibition	11.58536585	−10.34482759	4.62962963	5.483870968 ns	13.8122 **
**EOCS** **5 mg/kg bw**	Paw volume	1.50 ± 0.07	1.93 ± 0.01	2.13 ± 0.01	2.64 ± 0.06	2.93 ± 0.01
	Inhibition	8.536585366	−10.91954023	1.388888889	14.83870968	19.0608 **
**EOCS** **10 mg/kg bw**	Paw volume	1.51 ± 0.06	1.85 ± 0.03	1.98 ± 0.09	2.35 ± 0.10	2.55 ± 0.10
	Inhibition	7.926829268	−1.724137931	8.333333333	24.19354839	29.558 ***

EOCS: Essential oil *Cannabis sativa*. The results are presented as mean ± SD (n = 6); ‘*’ vs. Control ‘*’: *p* < 0.05; ‘**’ *p* < 0.01; ‘***’ *p* < 0.001; ns: not significant.

**Table 3:** Effect of CSEO and indomethacin on CRP plasma levels in rats. The results are presented as mean ± SD (n = 6).

	Day	Control	CFA	CFA + Indomethacin 3 mg/kg	CFA + EOCS 2.5 mg/kg	CFA + EOCS 5 mg/kg	CFA + EOCS 10 mg/kg
**CRP (mg/mL)**	Day 15	2.43 ± 0.14	4.2 ± 0.75 **	2.45 ±0.27	3.5 ± 0.37 *	3.2 ± 0.53	2.50 ± 0.12
	Day 25	2.55 ± 0.21	5.1 ± 0.68 ***	2.30 ± 0.83	2.70 ± 0.30	2.57 ± 0.37	2.33 ± 0.72

CFA: Freund’s complete adjuvant, INDO: indomethacin, EO: essential oil. Statistical analyses were done according to two-way ANOVA, followed by Tukey’s post hoc test, ‘*’ vs. control, *p* < 0.05; ‘**’ *p* < 0.01; ‘***’ *p* < 0.001.

## Data Availability

All data reported in this study are available within the article.
